# Prevalence and Fetomaternal Outcome of Placenta Previa at Salmaniya Medical Complex, Bahrain

**DOI:** 10.7759/cureus.27873

**Published:** 2022-08-11

**Authors:** Fatema Alhubaishi, Naeema Mahmood

**Affiliations:** 1 Obstetrics and Gynecology, Salmaniya Medical Complex, Manama, BHR

**Keywords:** blood transfusion, hysterectomy, lower segment cesarean section, hemorrhage, placenta previa

## Abstract

Background

Placenta previa is a condition which occurs when the placenta implants in the lower uterine segment, thus obstructing delivery. It is considered a grave pregnancy complication as it is associated with massive maternal hemorrhage. The condition is associated with previous cesarean delivery, multiple gestations, and increased maternal age. The placental villi may abnormally adhere, invade, or penetrate the myometrium causing accreta, increta, or percreta, respectively. It is the most common indication of peripartum hysterectomy. The gold standard for diagnosis of placenta previa is transvaginal ultrasound.

Objective

This study aims to calculate the prevalence of placenta previa in relation to the known risk factors and to determine the fetomaternal outcome which will aid in improving the obstetric care of patients with placenta previa.

Methods

A total of 216 placenta previa cases diagnosed between October 2014 and December 2018 were evaluated in a retrospective cross-sectional study. Analysis of the data was conducted using SPSS software, version 20 (IBM Corp., Armonk, NY).

Results

The total number of deliveries during the study period was 25,693 out of which 216 were diagnosed with placenta previa. Thus, the prevalence of placenta previa is 0.84%. The mean age at diagnosis was 32.8 years. At diagnosis, 23.1% of the cases were primiparous. Of the 216 patients, 1.9% were diagnosed with placenta percreta, of which 5.1% received a hysterectomy; 59.7% had uncomplicated elective cesarean delivery at 37-38 weeks of gestation. The mean gestational age at emergency delivery was 35.97 (+-3.1).

Conclusion

The study highlights that although risk factors increase the likelihood of placenta previa, it is necessary to rule it out in women with no known risk factors.

## Introduction

Placenta previa is a condition in which the placenta partially or wholly blocks the cervix thus obstructing delivery [1]. It carries the risk of severe obstetric complications including severe maternal hemorrhage, shock, fetal hypoxia, and death [2]. Risk factors for this condition include multiparity, previous abortion, previous cesarean delivery, multiple gestations, smoking, and increased maternal age [3]. Adverse infant outcomes include prematurity, stillbirth, and neonatal death [4]. The gold standard investigation for diagnosis of placenta previa is a transvaginal ultrasound. Placenta previa is diagnosed when the placenta is implanted less than 2 cm away from the cervical os. Women with placenta previa must be delivered by cesarean section; this is performed under regional or general anesthesia [5]. Availability of blood products at the institution where delivery is performed is mandatory as the condition can be associated with massive blood loss [6]. One in 200 deliveries is complicated by placenta previa; it is a leading cause of second and third trimester vaginal bleeding. The rate of placenta previa is increasing mainly due to the increase in the rate of cesarean section [7]. However, 0.3%-0.5% of cases are noted without a prior cesarean delivery [8].

Placenta accreta is a condition that often occurs along with placenta previa. In this condition, the placental villi abnormally adhere to the uterine myometrium due to the absence of decidua basalis, either partially or completely, causing life-threatening hemorrhage at the time of delivery, often leading to hysterectomy [9]. Placenta accreta is graded according to the depth of invasiveness: accreta, when the villi adhere to the myometrium without invading it; increta, when the villi invade the myometrium; and percreta, when the invasion reaches the uterine serosa [10]. The incidence is one in 533 pregnancies. It has been documented that 90% of women diagnosed with placenta previa or accreta are at an increased risk of antepartum hemorrhage prior to a gestational age of 37 weeks. The majority of them require an emergency preterm delivery. Hence, a scheduled delivery is preferably planned at 36-37 gestation [11]. The exact pathophysiology of why placenta previa occurs is not yet understood, however, there seems to be a dose-dependent relationship between endometrial damage, uterine scarring, and consequent placenta previa [12]. The most common presenting complaint is painless antepartum hemorrhage in the third trimester which ranges from mild to severe. The exact cause of the bleeding is not well understood. However, placental separation due to uterine contractions, cervical effacement/dilation, and advanced maternal age are contributing factors [8]. Furthermore, placenta praevia and accreta are mainly located in the lower segment, a location that predisposes to persistent uterine bleeding due to the development of new vessels and because it is an area of the uterus with poor contractility. Bleeding, in turn, could lead to an increased risk of blood transfusion, hysterectomy, ICU admission, thrombophlebitis, and maternal death [13].

The use of routine obstetric ultrasound during antenatal follow-up helps in the early diagnosis of placental abnormalities and hence, possible prevention of morbidity and mortality. Moreover, cases diagnosed at mid pregnancy should be repeated at approximately 32 weeks gestation, because the placenta undergoes a process termed trophotropism. Trophotropism occurs when the placenta grows into an area with increased blood supply, typically the fundus, and the part that is closest to the cervix eventually regresses and atrophies [14]. The complexity of placenta previa can be determined by tissue destruction, newly formed vessels, and vascular invasion of surrounding tissues [15]. Prenatal diagnosis of abnormal placentation allows the development of a multidisciplinary approach to achieve the best outcomes for mother and baby [16]. However, performing a transvaginal ultrasound routinely on all women is not recommended [17].

In cases where placenta accreta is diagnosed, placental magnetic resonance imaging is an accurate method of topographic stratification that makes it possible to define anatomy, plan the surgical approach, and consider other therapeutic possibilities [18]. In terms of management of placenta accreta, there are two treatment options: cesarean hysterectomy or a conservative approach. With the latter, there is a choice between leaving the placenta in situ and waiting for its later resolution, or a one-step surgery that addresses the abnormally invasive placenta, vascular control, and myometrial damage in a single surgical procedure [19,20].

The aim of the study was to determine the prevalence of placenta previa in Salmaniya Medical Complex and to examine its relation to various risk factors. We aim to raise awareness about the condition and its outcomes, highlighting the morbidity of the disease in terms of hysterectomy. We aim to highlight the seriousness of placenta previa and emphasize the importance of early diagnosis and prompt intervention. 

## Materials and methods

A retrospective cross-sectional study was conducted in the Obstetrics and Gynecology department of Salmaniya Medical Complex, in the Kingdom of Bahrain. The study was approved by the hospital’s ethics committee. All cases of placenta previa diagnosed between October 2014 and December 2018 at Salmaniya Medical Complex were included. These patients were diagnosed with placenta previa via ultrasound and further confirmed during cesarean section. All suspected cases of placenta previa had a repeat ultrasound examination at 32 weeks gestation for confirmation of the diagnosis. Gestational age was calculated by the last menstrual period and by the first ultrasound. These patients were evaluated for the presence of risk factors such as previous cesarean section, parity, and maternal age. They were also evaluated for the occurrence of complications during cesarean section including bleeding and cesarean hysterectomy. Gestational age at delivery and neonatal birth weight were recorded. The SPSS software, version 20 (IBM Corp., Armonk, NY) was used for statistical analysis. The descriptive analysis that was utilized included the mean, standard deviation, and frequency distribution.

## Results

The total number of deliveries during the study period was 25,693. A total of 216 cases were diagnosed with placenta previa. Thus, the prevalence of placenta previa during the study period was 0.84%. The mean age of participants was 32.88 (+-5.89), as seen in Table 1.

**Table 1:** Maternal demographic data: age

Age in years	Number of patients	Percentage	Mean	Standard Deviation
18-25	22	10.1%	32.88	+-5.892
26-35	124	57.4%		
36-45	64	29.6%		
>45	6	2.7%		

The majority (74.1%) of the patients were Bahraini nationals; 23.1% (50 patients) were primigravida while 22.2% (48 patients) were para 1 and 25.9% (56 patients) were para 2 (Table 2).

**Table 2:** Maternal demographic data: parity

Parity	Number of Patients	Percentage
Primi	50	23.1
Para 1	48	22.2
Para 2	56	25.9
Para 3 or more	62	28.7

Of the 216 cases, 96% had placenta previa while 1.9% had placenta percreta. Three cases were diagnosed with placenta accreta and one with placenta increta (Table 3).

**Table 3:** Type of placental abnormality diagnosed by transvaginal ultrasound

Placental abnormality	Placenta previa	Placenta accreta	Placenta increta	Placenta percreta
Frequency	208	3	1	4
Percentage	96.2%	1.3%	0.5%	1.9%

In terms of risk factors, 72.7% of women diagnosed with placenta previa in our study had no previous cesarean deliveries (Table 4).

**Table 4:** Maternal demographic data: number of previous cesarean deliveries

Number of cesarean sections	Number of Patients	Percentage
No previous cesarean sections	157	72.7
Previous 1 cesarean section	29	13.4
Previous 2 cesarean sections	23	10.6
Previous >3 cesarean sections	7	3.2

The worldwide incidence of placenta previa in women with no previous cesarean deliveries is 0.3%-0.5% [11]. In our study, however, the incidence of placenta previa in women with no previous cesarean was 0.72%. When examining management pathways, 59% of the cases had elective cesarean deliveries, scheduled between 37-38 weeks of gestation, while 41% underwent emergency cesarean deliveries due to antepartum hemorrhage, at an average of 35.2 weeks of gestation, with a mean birth weight of 2.1 kg. For the management of intraoperative hemorrhage, Bakri balloon (Cook Women's Health, Spencer, IN, USA) insertion was performed in 4.6% of cases, 0.9% of cases needed bilateral iliac artery ligation, and 5.1% received a hysterectomy. Of the 5.1% that received a hysterectomy, 27.2% were diagnosed with placenta previa antenatally, while 72.7% were diagnosed with placenta accreta, increta or percreta during cesarean section. In histopathology, 33% were confirmed as accreta, 75% were confirmed as increta, and 25% were confirmed as percreta. All cases of the placenta accreta spectrum were managed by hysterectomies in comparison with 1.4% of the placenta previa cases. Furthermore, the fetomaternal outcome of cases in the placenta accreta spectrum is comparable. No maternal mortality was noted in the study. One case of intrauterine fetal death was noted and another case of intrauterine growth restriction. Of the 216 deliveries, 43 babies were handled by the NICU team for prematurity (Table 5). No other fetal complications were noted.

**Table 5:** Fetomaternal outcome

Fetomaternal outcome	Percentage
Scheduled term delivery	59%
Emergency Preterm delivery	19.9%
Intrauterine growth restriction	0.5%
Intrauterine fetal death	0.5%

## Discussion

This study found that the prevalence of placenta previa (0.84%) is higher than the worldwide prevalence of 0.3%-0.5% [21]. There is generally an increase in the rate of placenta previa worldwide, mainly due to the increase in the cesarean section rate [22]. However, in contrast to worldwide trends, our study found that 72.7% of cases were primigravida at diagnosis. In addition, maternal age was not found to be a significant risk factor. This point illustrates that additional risk factors may have a larger impact on the incidence of placenta previa other than the originally described risk factors of advanced maternal age, previous cesarean section, and parity.

A meta-analysis study showed that the rate of placenta previa is affected by regional differences; it is higher among Asian countries (1.22%) and lower in Europe (0.36%), North America (0.29%), and sub-Saharan Africa (0.27%) [2]. However, It was not clear whether those differences were due to ethnicity or due to better diagnostic methods [23,24]. Massive obstetric hemorrhage in placenta previa is associated with severe maternal morbidity and mortality worldwide, accounting for 30% of maternal deaths in Asia [25]. No case fatalities were reported in this study. The maximum number of blood donated to a patient in our study was 8 units with a mean of 2.5 units. This indicates that liberal blood transfusion and cesarean hysterectomy are important factors in reducing the case-fatality rate in women with placenta previa/accreta. 

A cohort study involving 3,550,842 deliveries was conducted comparing the outcome of deliveries involving mothers with placenta previa after 37 weeks of gestation to those without placenta previa. It was found that placenta previa was an independent risk factor for adverse neonatal outcomes [26]. However, it is reasonable to say that the presence of placenta previa itself increases adverse neonatal outcomes when delivered at term. The reasons behind that are unknown. Some debate that placenta previa is not an independent risk factor for impaired fetal growth with no significant difference in birth weight in neonates born to mothers with placenta previa, and those delivered to normal placenta locations [27,28].

The shortcomings of this study include the lack of analysis of other possible risk factors such as smoking status, body mass index, and infertility treatment. 

## Conclusions

The morbidity of the condition in terms of hysterectomy is significant. We emphasize the importance of placental localization by ultrasound routinely to all antenatal patients, regardless of risk factors. The use of routine obstetric ultrasound in antenatal follow-up helps in the early diagnosis of placental abnormalities and hence, the possible prevention of morbidity and mortality. 
